# Haplotype diversity of Heterodera koreana (Tylenchida: Heteroderidae), affecting bamboo in Korea

**DOI:** 10.21203/rs.3.rs-3205194/v1

**Published:** 2023-09-08

**Authors:** Heonil Kang, Hyoung-Rai Ko, Yeon-Jeong Lim, Eun-Hyeong Park, Eun-Hwa Kim, Se-Keun Park, Byeong-Yong Park, Hyerim Han

**Affiliations:** National Institute of Forest Science; National Institute of Agricultural Sciences; National Institute of Forest Science; National Institute of Agricultural Sciences; National Institute of Agricultural Sciences; National Institute of Agricultural Sciences; National Institute of Agricultural Sciences; National Institute of Forest Science

**Keywords:** Afenestrata group, Bamboo, COI, Haplotype, Heterodera koreana

## Abstract

In a survey of plant-parasitic nematodes in agricultural fields, cyst-forming nematodes were found in soil planted bamboo in Korea. The aim of this study was to identify the cyst nematodes based on morphological and molecular characteristics. As the results, the morphology and morphometrics of cysts and second-stage juveniles (J2s) were consistent with those of previous descriptions of *Heterodera koreana*. In phylogenetic analyses based on DNA sequences, these cyst nematodes were clustered together with clade of *H. koreana* in internal transcribed spacer (ITS) region, and large subunit D2-D3 segments (LSU D2-D3). These nematodes were clustered together with clade of *H. koreana* in cytochrome c oxidase subunit I (*CO*I) gene, but a haplotype was different when compared with previous reported haplotypes (haplotype A-C) in Japan. This study showed these cyst nematodes were identified as *H. koreana*, and a new haplotype of *H. koreana* is distributed in Korea. We suggest that the new haplotype of *H. koreana* name as haplotype D.

## Introduction

Plant-parasitic nematodes (PPNs), which are over 4,100 species, have interacted with their hosts (Decraemer and Hunt, 2006; Jones et al., 2013). Damage caused by PPNs has been estimated at 157 billion USD per year (Nicol et al., 2011). Among the PPNs, cyst nematodes are regarded as most important PPNs worldwide, and contain eight genera (as of 2021). Economic damages in many plants occurred by two genera, *Heterodera* and *Globodera*. The genus *Heterodera* is one of the most important PPNs worldwide, and are described as about 85 species including soybean cyst nematode (*Heterodera glycines). Heterodera* species reported in Korea are as follows: soybean cyst nematode (*H. glycines*) on soybean in 1983 (Choi and Choi, 1983), Korean cyst nematode (*H. koreana*) on bamboo in 1992 (Vovlas et al., 1992), sugar beet cyst nematode (*H. schachtii*) on Chinese-cabbage in 2011 (Kim et al. 2016), white soybean cyst nematode (*H. sojae*) on soybean in 2016 (Kang et al., 2016), clover cyst nematode (*H. trifolii*) on Chinese-cabbage in 2017 (Mwamula et al., 2018), and rice cyst nematode (*H. oryzae*) on rice in 2020 (Mwesige et al., 2020).

*H. koreana* and *H. sojae* were first discovered in Republic of Korea, and were reported as a novel cyst nematode species. Since then, the nematodes distributions were reported in other countries (Kang et al., 2016; Vovlas et al., 1992). *H. koreana* was described as *Afenestrata koreana* at the first time by Vovlas et al. (1992), but the genus *Afenestrata* was subsequently changed to *Heterodera* (Mundo-Ocampo et al., 2008). Because the genus *Afenestrata* was not classified as a specific clade and was clustered together with *Heterodera* spp. in phylogenetic study, synonymisation of *Afenestrata* with *Heterodera* was proposed (Mundo-Ocampo et al., 2008). Recently, the bamboo is cultivated as crop for tea, seasoned vegetables in Korea, and damage by the Korean cyst nematode is expected. Since the Korean cyst nematode (*H. koreana*) firstly reported, there were no further findings or studies of the cyst nematode. The study of the cyst nematode based on molecular characteristics is required because it is difficult to identify the *Heterodera* species using morphological and morphometrical characteristics.

In this study, we identified the cyst nematodes extracted from bamboo using the morphological characteristics and the molecular characteristics based on DNA barcoding gene such as LSU D2-D3 expansion segments, ITS region, and mitochondrial DNA *CO* gene. Furthermore, we specialized the haplotype of the Korean cyst nematode based on the *CO*I gene and named the new haplotype.

## Materials and Methods

### Nematode isolation.

Soil samples were collected from bamboo rhizosphere of three fields a depth of approximately 15 cm soil in Sacheon city, Gyeongsangnam-do in Korea using soil sampler (diameter 2 cm). The samples were labeled as BC348, DR437, and DR1256, respectively (Table 1). Cysts were extracted by sieving method using 20 and 60 mesh sieves (Kang et al., 2016). After extraction, cysts and J2s were transferred into a watch glass containing tap water using forceps and pipette under a stereomicroscope (MZ205; Leica, Wetzlar, Germany) and were kept at 4°C until further use.

### Morphological analysis.

For light-microscopic observations, J2s were killed and fixed by addition of 80°C FG 4:1 fixative (Southey, 1986). The nematodes were fixed for at least 24 hr, then processed according to a Seinhorst method (Cid Del Prado Vera and Subbotin, 2012; Seinhorst, 1959). Specimens were mounted on Cobb slides and sealed with a paraffin ring and glycerin (Cobb, 1917). Vulval cones were cut under a stereomicroscope (M205; Leica, Wetzlar, Germany), and were transferred to glycerin on slide glasses. The nematodes were observed, measured, and photographed with the aid of a compound microscope (DM5000; Leica, Wetzlar, Germany) equipped with microscope digital camera (DFC450; Leica, Wetzlar, Germany). Also, overall shape of cysts, shape of annule, vulval cone, head and lateral field were observed. The nematodes were identified morphologically based on *Heterodera* species identification key authored by Subbotin et al. (2010).

### Molecular analysis.

To extract genomic DNA, each single cyst of three different populations was transferred to a slide-glass on a small drop of distilled water, opened and its contents crushed using a filter paper chip (2 mm × 2 mm) and forceps. Using forceps, the chip having crushed eggs and J2s was transferred into a PCR tube containing 30 μl lysis buffer (sterilized triple distilled water, 1 M Tris-HCl, 10% Triton-X 100, 100 μg/ml Proteinase K, 2 M KCl, 1 M MgCl_2_) for extracting the nematode DNA (modified Iwahori et al. 2000). The tubes were incubated in a Thermal cycler (PTC-200, MJ Research, Alameda, CA, USA) at 60 °C for 1hr and 94 °C for 10 min.

Two ribosomal RNA fragments, i.e. the LSU D2-D3 segments, ITS regions, and *CO* gene of mitochondrial genome were amplified. Primers for D2-D3 segments amplification were D2A (5’-ACAAGTACCGTGAGGGAAAGTTG-3’) and D3B (5’-TCGGAAGGAACCAGCTACTA-3’) (Subbotin et al., 2006). Primers for ITS amplification were TW81 (5’-GTTTCCGTAGGTGAACCTGC-3’) and AB28 (5’-ATATGCTTAAGTTCAGCGGGT-3’) (Subbotin et al., 2000). A primer set of JB3 (5’-TTTTTTGGGCATCCTGAGGTTTAT-3’) and JB5 (5’-AGCACCTAAACTTAAAACATAATGAAAATG-3’) for *CO* gene was used in the PCR reaction (Derycke et al., 2005).

The PCR condition was as follows: pre-denaturation stage; 94°C for 5 min, cycling stage (n = 40); denaturation at 94°C for 1 min, annealing at 56°C (D2-D3 segments), 58°C (ITS region), and 57°C (*CO* gene) for 1 min, respectively, and extension at 72°C for 2 min. The final extension was continued at 72°C for 10 min. In order to verify the PCR amplicon, electrophoresis was performed using 0.5x TAE buffer on 1% agarose gel. The amplicon was subsequently purified using commercial PCR Purification Kit (Qiagen, Valencia, CA). All strands of the PCR amplicons were cycle-sequenced with an ABI PRISM BigDye Terminator version 1.1 Cycle Sequencing Kit and electrophoresed in each direction on an ABI Prism ABI 377 Genetic Analyzer (PE Applied Biosystems, USA). The newly obtained sequences were submitted to the GenBank database (Table 1).

### Phylogenetic analysis.

For phylogenetic study, the sequences of three populations compared with GenBank nematode sequences using the BLAST homology search program. The closest sequences were selected for phylogenetic analyses. Outgroup taxa for each dataset was chosen according to previous phylogenetic study for cyst-forming nematodes (Mwesige et al., 2020; Kang et al., 2016), *Cryphodera brinkmani* Karssen & van Aelst, 1999 and *Meloidogyne alni* Turkina & Chizhov, 1986 (De Luca et al., 2013; Subbotin et al., 2000). The newly obtained and published sequences for each gene were aligned using Clustal W with default parameters (Thompson et al., 1994). Sequence alignments were manually edited using BioEdit (Hall et al. 1999). The alignment quality was examined by naked-eyes, and optimized manually by adjusting the ambiguous nucleotide positions. Models of base substitution were evaluated using MODELTEST3.7 combined with PAUP4.0 (Huelsenbeck and Ronquist, 2001; Posada and Crandall, 1998; Swofford, 2003). The Akaike-supported model, the base frequency, the proportion of invariable sites, and the gamma distribution shape parameters and substitution rates in the AIC were then used in phylogenetic analyses. Bayesian analysis was performed to confirm the tree topology for each gene separately using MrBayes 3.1.2 running the chain for 1 × 10^6^ generations and setting the ‘burn-in’ at 2500 (Huelsenbeck and Ronquist, 2001). The MCMC (Markov Chain Monte Carlo) method was used within a Bayesian framework to estimate posterior probabilities of the phylogenetic trees (Larget and Simon, 1999), and generate a 50% majority- rule consensus tree. The posterior probabilities are given on appropriate clades. Trees were visualized using TreeView (Page, 1996).

### Data of COI gene analysis.

The sequences of the *CO*I gene were assembled and aligned using MEGA version X (Kumer et al., 2018) with accession numbers LC202153–93, MW642452–4 and OL813218–9. DnaSP version 5.10.1 (Librado and Rozas, 2009) was used to carry out preliminary analyses of nucleotide polymorphism and haplotype variation, and to generate Arlequin haplotype files. We used PopART (Leigh and Bryant, 2015) to construct and examine median-joining haplotype networks (Bandelt et al., 1999), and Arlequin version 3.5.2.2 (Excoffier and Lischer, 2010) to estimate haplotype diversity and nucleotide diversity, and to test for genetic structure with an analysis of molecular variance (AMOVA).

## Results

### Morphological analysis.

**Figure 1:**
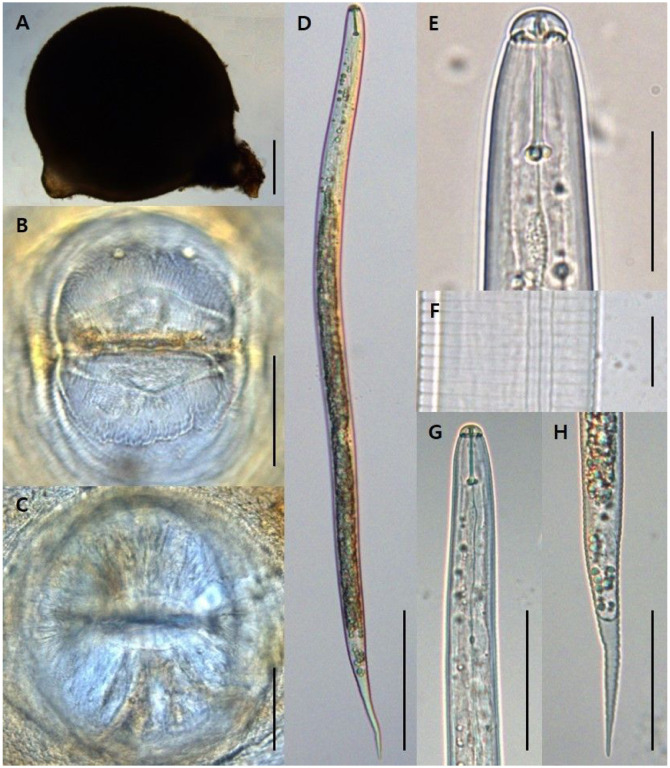
The morphological characterization of cyst, vulval cone and second-stage juvenile of *Heterodera koreana*. A: Lemon-shaped cyst; B-C: Vulval cone; D: Entire body of second-stage juveniles; E: Head region of second-stage juveniles; F: Lateral field; G: Anterior of second-stage juveniles; H: Tail region. (Scale bars: A = 100 μm, B-C = 50 μm, D = 100 μm, E = 20 μm, F = 10 μm and G-H = 50 μm.)

Morphological characters and morphometric features of cysts, vulval cone of cysts and J2s were examined and measured for species identification. Lemon-shaped cysts, which are variable in size (377–903 μm) with distinct neck and vulval cone, were observed (Fig. 1A). Cuticle appeared light to dark brown in colour. Stylet and other pharyngeal structures were indistinct. Gelatinous egg sac was not observed and the cyst cuticle has irregular zigzag pattern on mid-body. Vulval cone with lacking-fenestration was observed and covered with tuberculate pattern (Fig. 1B and 1C). J2s had a cylindrical body, tapering posteriorly, straight of slightly ventrally curved after fixation (Fig. 1D). Stylet with a length of 20–21 μm was well developed, stylet-knobs were oviform (Fig. 1E and 1G), and the body length ranged from 454 to 545 μm (Table 2). The J2s had three incisures in the lateral field (Fig. 1F). Anus and hyaline part of tail were distinct, and hyaline terminal section was average 50.9 (45.5–55.0) μm long (Fig. 1H).

### Molecular and phylogenetic analysis.

The LSU D2-D3 segments, ITS region, and *CO*I gene of the mtDNA were amplified as indicated in methodology section. The sequenced LSU D2-D3 segments, ITS region, and *CO*I are 751–755, 902–908, and 424–425 bp, respectively. A BLASTn search on the LSU D2-D3 segments and ITS region revealed similarities with the Afenestrata group of *Heterodera* species such as *H. Koreana* and *H. hainanensis*. The highest match of LSU D2-D3 segments sequences was H. koreana (LC202092), with 100% identities and no gaps. The ITS region results also revealed the most similar species with *H. koreana* (KX640828), with 99.89% identities (901/902) and no insertions/deletions. In addition, a BLASTn search of *H. koreana* on the *CO*I revealed high-scoring matches with *H. koreana* (LC202153), which is the species isolated from *Phyllostachys nigra var*. *henonis* in Iwate in Japan. The identities between the Korean population (this study) and *H. koreana* (LC202153) were 98.21% (385/392), with no insertions/deletions.

**Figure 2:**
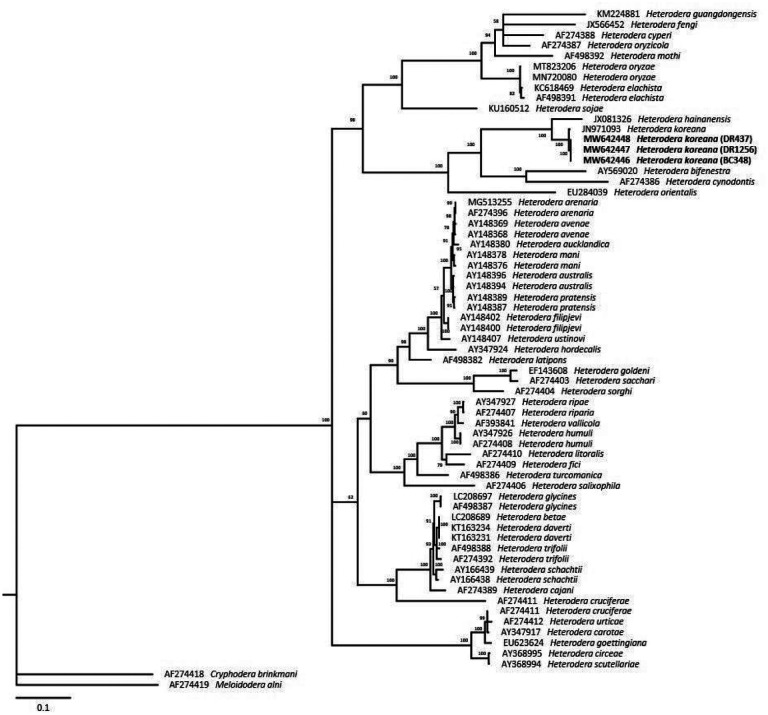
Phylogenetic relationships within population and species of *Heterodera*. Bayesian 50% majority rule consensus tree from two runs as inferred from the analysis of the D2-D3 of 28S rDNA gene sequences under the GTR+I+G model. Posterior probability values more than 50% are given in appropriate clades. Newly sequenced samples are indicated by bold font.

**Figure 3:**
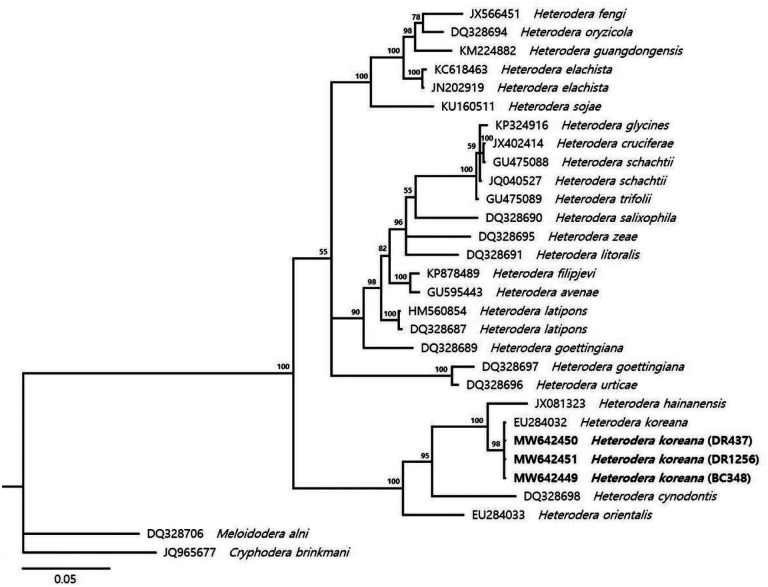
Phylogenetic relationships within population and species of *Heterodera*. Bayesian 50% majority rule consensus tree from two runs as inferred from the analysis of the ITS rRNA gene sequences under the TVM+I+G model. Posterior probability values more than 50% are given in appropriate clades. Newly sequenced samples are indicated by bold font.

**Figure 4:**
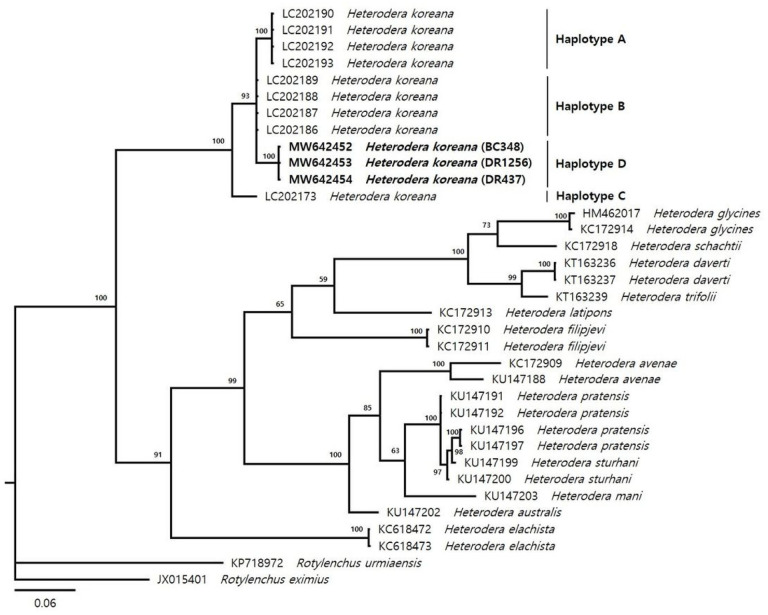
Phylogenetic relationships between Heterodera species. Bayesian 50% majority rule consensus tree as inferred from the analysis of the *CO* gene sequence alignment under the TVM+I+G model. Posterior probabilities over 50% are given for appropriate clades. Newly obtained sequences are indicated by bold font.

The molecular phylogenetic relationships of Korean populations of *H. koreana* were shown in Figs. 2, 3, and 4. The phylogenetic tree of the LSU D2-D3 segments region dataset of *Heterodera* species is shown in Fig. 2. The average nucleotide compositions were as follows: 19.33% A, 21.47% C, 33.92 G and 25.28% T. Using *Cryphodera brinkmani* and *Meloidodera alni* as the outgroup taxa, the molecular phylogeny strongly supported monophyly of *Heterodera* species. Phylogenetic tree inferred from ITS region dataset was in Fig. 3. The average nucleotide compositions were as follows: 19.17% A, 22.44% C, 29.04% G and 29.35% T. Three Korean populations of *Heterodera* species were close to *H. koreana* when *C. brinkmani* and *M. alni* as the outgroup taxa were used. The results showed that BC348, DR437, and DR1256 population belong to ‘*Afenestrata’* group clade.

Phylogenetic tree based on *CO*I gene of mtDNA was described in Fig. 4. The average nucleotide compositions were as follows: 24.50% A, 8.68% C, 14.18 G and 52.64% T. Using *Rotylenchus eximius* and *R. urmiaensis* as the outgroup taxa, the molecular phylogenetic relationship of the dataset, which contains three Korean populations and previously registered data in NCBI, was closed to *H. koreana*. However, three Korean populations were not clustered together with Japanese haplotype of *H. koreana*, haplotype A-C, and were classified to a new clade of *H. koreana*.

### Data of COI gene analysis.

**Figure 5:**
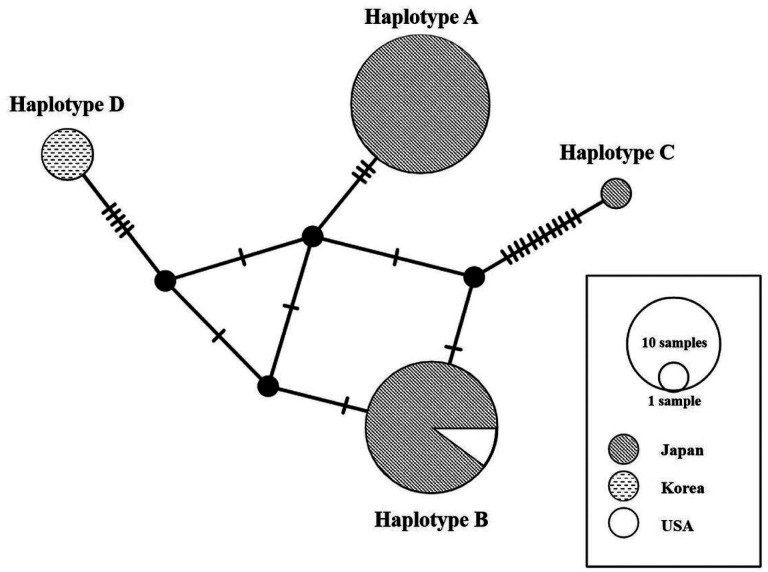
Median-joining haplotype network. Haplotypes mentioned in text are indicated. Black circle represents an un-sampled haplotype. Mutations are indicated with hatch-marks.

We identified four haplotypes (Haplotype A–C, and Korean haplotype) in the 46 *CO*I sequences (357 bp), and the dataset included 21 polymorphisms (Table 3). The network analysis showed a radial-shaped haplotype network with the most common Haplotype A (48%) and B (43%) occupying a central position with the rest of the haplotypes radiating differing from it by up to 13 substitutions (Fig. 5). Haplotype A and C were found in Japan. Haplotype B was found in both Japan and USA. The Korean haplotype was newly found in this study in Korea. We analyzed haplotype diversity and nucleotide diversity by regional populations. In Japan populations, the haplotype diversity was 0.5317 ± 0.0319 and the nucleotide diversity was 0.0090 ± 0.0053. The Tajima’s D statistic was negative for Japan populations (−0.68579). The Korean and USA populations were no diversities in the haplotype and the nucleotide, because there was no variation in regional populations.

Pairwise *F*_*ST*_, which is a fixation index between regional populations, was calculated to estimate in genetic differentiation that can be caused by the genetic structure. The Korean population had a high fixation index value 1.000 with USA populations and 0.680 with Japanese populations, indicating a differentiated genetic structure to the Korean population. On the other hand, the Japanese and USA populations had a low differentiation with a fixation index of 0.177. The AMOVA showed that variation by region was responsible for 60.46% of the total variation. The remaining 39.54% of the variance was explained by the variation among populations within region (7.76%) and variation within populations (31.78%) (Table 4).

## Discussion

During a PPNs survey in 2020, three populations of *Heterodera* species were isolated from rhizosphere of bamboo in Republic of Korea. Three cyst-forming nematodes were identified as *H. koreana* using morphological identification key for *Afenestrata* sensu stricto group and phylogenetic analysis (Table 2, Figs. 1 and 2). The genus *Heterodera* species contains seven sensu stricto groups, which are *Afenestrata, Avenae, Cyperi, Goettingiana, Humuli, Sacchari* and *Schachtii* group (Subbotin et al. 2010). Bayesian trees inferred from LSU D2-D3 segments and ITS region showed *H. koreana* is related to *Afenestrata* group (Figs. 2 and 3). *Afenestrata* group includes the following seven species, which are *H. africana*, *H. axonopi, H. bamboosi, H. hainanensis, H. koreana, H. orientalis* and *H. saccharophila* (Mundo-Ocampo et al., 2008; Zhuo et al., 2013). Among them, three *Heterodera* species, which are H. bamboosi, H. koreana and *H. hainanensis*, has been recorded from bamboo in the world (Kaushal and Swarup, 1988; Vovlas et al., 1992; Zhou et al., 2013), but the sequences of *H. bamboosi* were absent in GenBank. Nevertheless, *H. koreana* could be distinguish from *H. bamboosi* by the shorter body length in J2 (446 vs. 472 μm), vulval cone present in female (Subbotin et al., 2010).

Morphology and morphometrics of the cysts and the J2s of three Korean populations were consistent with the described *H. koreana* in China (Wang et al., 2012). However, these populations differed from original descriptions in Republic of Korea and Japanese population by the shorter cyst body length, the longer J2s body length, tail length, and length of hyaline region (Sekimoto et al., 2017; Vovlas et al., 1992). The ‘c’ value and ‘tail length’ in Korean populations were inconsistent with the original description of Iranian population (Maafi and Tehari, 2015). These differences in morphology could be explained as a result of intraspecific variation (Wang et al., 2012).

Recently, molecular and phylogenetic analysis based on the DNA barcoding genes such as LSU D2-D3 segments, ITS region, and mtDNA *CO*I gene are very important to speed up and simplify the identification of animals including plant-parasitic nematodes. The *CO* gene is very powerful DNA barcoding marker, and has been used in barcoding of *Heterodera* species since 2005 (Blok and Powers, 2009; Derycke et al., 2005; Subbotin et al., 2015; Vovlas et al., 2015). However, our phylogenetic study based on the *CO*I showed the haplotype of the Korean populations was not clustered together with Japanese populations (Fig. 4).

In previous study, three *CO* haplotypes of *H. koreana* were found in Japan, and named as haplotypes A, B and C (Table 3). The haplotype A is dominant in Japan (Sekimoto et al., 2017). However, our study showed the new haplotype of *H. koreana* was present in Korea because the haplotype of the Korean populations was distinguished from Japanese haplotype, and the AMOVA analysis also showed that the regional differences (60.46%) between Korean populations and Japanese population was greater than that between total populations (31.78%) (Table 4). Thus, we suggest that the new haplotype of *H. koreana* is present in Republic of Korea and name as haplotype D (Table 3 and Fig. 5).

To investigate associations between genetic haplotype and phenotype in the nematodes, we performed morphological comparisons between the Korean haplotype and the haplotypes reported in China and Japan. Our study showed that the whole length and hyaline portion in J2s in the Korean populations were similar to that of the Chinese population (Wang et al., 2012) (Table 2). However, our results showed that the Korean population had a longer J2 body length (499 ± 15.5 vs. 458 ± 17.3 μm) and a longer hyaline length of the J2 tail (51 ± 3.0 vs. 41 ± 3.2 μm) than those of the Japanese population (Sekimoto et al., 2017) (Table 2). Thus, we suggest that the differences in the haplotype of the *CO* gene sequence may be affect to the morphological differences within the species, *H. koreana*. The studies on the association between genotype of LSU D2-D3, ITS regions, *CO*, and morphological characteristics were conducted in marine nematodes. The results showed that highly divergent genotype clusters were accompanied by morphological differences (Derycke et al., 2008), and in the *CO* gene and ITS region particularly (Fonseca et al., 2008). Because the sequence of the Chinese haplotype is absent in NCBI Genbank, the study on the association between genetic haplotype and phenotype is required. In addition, the various haplotypes could be distributed in Republic of Korea due to the similar morphology between original description of *H. koreana* in Republic of Korea and those of the Japanese population (Sekimoto et al., 2017; Vovlas et al.,1992). The haplotype may be associated with phenotypes of the nematodes like ecotype, and pathotype. Therefore, we require the study on correlations between haplotype and phenotype of the nematodes in further study.

## Figures and Tables

**Table 1:** Nematode samples used in this study

Population code	Collection date	Host plant	Coordinates	Accession No		
				LSU D2-D3	ITS	*CO*I
BC348	17th Dec. 2019	Bamboo	35.09973295964911, 128.0966177424182	MW642446	MW642449	MW642452
DR437	18th Dec. 2019	Bamboo	35.11422712938377, 128.1349239712546	MW642448	MW642450	MW642454
DR1256	17th Dec. 2019	Bamboo	35.10823722627927, 128.1225063270747	MW642447	MW642451	MW642453

**Table 2:** Morphometric comparison of *Heterodera koreana* populations from Republic of Korea, Japan, China and Iran. All measurements are in μm and in the form: mean ± s.d. (range).

Stage	Character	Korea (this study)			Korea	Japan	China	Iran
		BC348	DR437	DR1256	Vovlas et al., 1992	Sakimoto et al.,2017	Wang et al., 2012	Maafi and Tehari, 2015
Cyst	n	7	10	7	20	10	5	21
	L (including neck)	647.4 ± 117.9	611.6 ± 81.5	592.1 ± 139.5	840 ± 149.0	641 ± 65.8	748 ± 75.0	502 ± 70.0
		(509−903)	(489−709)	(377−844)	(630–1174)	(524–770)	(700–860)	(420–640)
	Body diam.	544.9 ± 100.9	521.4 ± 111.0	506.5 ± 102.3	520 ± 90.0	574 ± 60.7	680 ± 63.0	408 ± 60.0
		(442.5−713.9)	(326.6−620.1)	(316.5−605.9)	(400–653)	(482–706)	(620–760)	(320–520)
	L/W ratio	1.2 ± 0.1	1.2 ± 0.2	1.2 ± 0.1	1.5 ± 0.4	1.1 ± 0.1	1.1 ± 0.1	1.2 ± 0.1
		(0.9–1.4)	(1.0–1.5)	(1.0–1.4)	(1.0–2.9)	(1.0−1.2)	(1.0−1.2)	(1.1−1.5)
Vulval cone	Vulval slit length	48.6 ± 2.1	52.3 ± 2.7	48.2 ± 2.4	49.0 ± 4.3	49.9 ± 4.9	48.8 ± 5.1	51.9 ± 4.3
		(46.0−51.6)	(48.4−56.4)	(44.0−51.9)	(42.0−56.0)	(40.5−59.8)	(41.3−55.0)	(46.0−59.0)
Second-stage Juvenile	n	10	10	10	20	20	20	14
	L	516.5 ± 17.7	487.9 ± 18.1	491.8 ± 10.6	446 ± 28.0	458 ± 17.3	513 ± 29.2	455 ± 11.3
		(480−545)	(454−514)	(476−507)	(390–509)	(424–483)	(448–553)	(437–472)
	a	26.5 ± 1.1	25.3 ± 1.3	25.7 ± 0.5	28.0 ± 1.7	26.3 ± 0.8	29.9 ± 1.6	29.9 ± 0.9
		(24.8−28.1)	(23.2−27.1)	(24.7−26.5)	(25.0−32.0)	(25.3−27.8)	(26.0−32.2)	(28.3−31.5)
	c	6.7 ± 0.2	6.6 ± 0.1	6.6 ± 0.1	6.7 ± 0.3	6.3 ± 0.2	6.7 ± 0.7	7.4 ± 0.9
		(6.2–6.9)	(6.4–6.8)	(6.4–6.8)	(6.1–7.3)	(6.0–7.0)	(6.2–8.1)	(6.0–8.9)
	Stylet	21.0 ± 0.5	20.7 ± 0.4	20.7 ± 0.3	18.0 ± 1.5	17.5 ± 0.6	19.0 ± 0.9	18.1 ± 0.5
		(20.5–21.8)	(20.2–21.1)	(20.3–21.0)	(16.0–20.0)	(16.4–18.9)	(17.5–20.5)	(17.0–19.0)
	Labial region height	4.7 ± 0.3	4.6 ± 0.5	4.7 ± 0.4		3.6 ± 0.2		3.0
		(4.4–5.2)	(3.9–5.4)	(4.0–5.2)		(3.3–3.9)		(3.0)
	Labial region diam.	8.7 ± 0.3	8.5 ± 0.2	8.5 ± 0.2		8.1 ± 0.2		7.5 ± 0.5
		(8.3–9.3)	(8.3–8.8)	(8.0–8.7)		(7.7–8.4)		(7.0–8.0)
	Anterior end to median bulb value	75.7 ± 1.2	71.8 ± 3.4	73.6 ± 2.0	72 ± 7.2	70 ± 3.0	72 ± 6.8	72 ± 1.7
		(73.0–77.1)	(63.8–75.6)	(70.677.3)	(65–98)	(65–77)	(60–80)	(70–75)
	Anterior end to excretory pore	110.4 ± 2.9	102.9 ± 7.4	105.8 ± 5.0	95 ± 4.3	105 ± 3.0	111 ± 6.7	100 ± 2.5
		(105.9–113.8)	(84.3110.3)	(93.0110.9)	(88–103)	(98–111)	(97–118)	(96–103)
	Body diam. at mid-body	19.5 ± 0.9	19.3 ± 1.0	19.2 ± 0.5	16.0 ± 1.0	17.4 ± 0.4	17.2 ± 0.5	15.2 ± 0.4
		(18.0–21.1)	(18.2–21.6)	(18.4 ~ 20.0)	(14.0–17.0)	(16.5–18.1)	(16.0–18.0)	(15.0–16.0)
	Body diam. at anus	13.7 ± 0.6	13.1 ± 0.3	13.2 ± 0.6	-	12.2 ± 0.4	-	10.1 ± 1.0
		(13.1–14.7)	(12.4–13.4)	(12.3–14.5)		(11.5–12.7)		(8.0–11.0)
	Tail length	76.7 ± 4.4	74.0 ± 3.3	74.6 ± 1.4	66 ± 4.4	72 ± 4.4	77 ± 8.9	62 ± 6.9
		(70.6–85.0)	(68.2–78.9)	(72.0–76.7)	(59–74)	(63–79)	(62–88)	(51–74)
	Hyaline region	52.4 ± 4.3	51.0 ± 1.8	49.3 ± 3.0	40 ± 2.5	41 ± 3.2	49 ± 5.2	44 ± 1.8
		(48.1–60.1)	(48.8–53.5)	(45.5–55.0)	(35–46)	(33–45)	(39–56)	(40–47)

**Table 3:** Variable position in the four *CO* haplotypes of Heterodera koreana populations.

Haplotype	Accession No.	Variable position (bp)																		
		33	45	57	72	75	81	87	132	159	165	171	186	189	192	199	213	228	257	267
A	LC202164–7	A	G	A	C	T	A	A	G	G	A	T	A	A	T	G	A	C	G	T
	LC202169–83	.	.	.	.	.	.	.	.	.	.	.	.	.	.	.	.	.	.	.
	LC202190–3	.	.	.	.	.	.	.	.	.	.	.	.	.	.	.	.	.	.	.
B	LC202153–63	A	G	A	T	G	A	A	A	G	A	T	A	A	T	G	T	T	G	T
	LC202168	.	.	.	.	.	.	.	.	.	.	.	.	.	.	.	.	.	.	.
	LC202184–9	.	.	.	.	.	.	.	.	.	.	.	.	.	.	.	.	.	.	.
	OL813218–9	.	.	.	.	.	.	.	.	.	.	.	.	.	.	.	.	.	.	.
C	LC202173	A	A	T	T	T	G	A	A	A	A	A	G	T	C	A	T	T	A	T
D	MW642452–4	G	G	A	T	A	A	G	G	A	G	T	A	A	T	G	T	T	G	C

**Table 4:** Analysis of molecular variance of three populations of *Heterodera koreana*. Group refers to the pooling of Japan and USA together and Korea alone.

Source of variation	d.f	Sum of squares	Variance components	Percentage of variation	Fixation indices	p-value^a^
Among group	1	18.887	2.76860	60.46	*F*_ct_ = 0.60460	0.00089
Among populations within groups	1	2.810	0.35515	7.76	*F*_SC_ = 0.68216	0.03317
Within populations	43	62.585	1.45547	31.78	*F*_ST_ = 0.19615	0.00000
Total	45	84.283	4.57922			

a Significance tests were performed with 10,100 permutations.
